# Epigenetic Modifiers Revamp Secondary Metabolite Production in Endophytic *Nigrospora sphaerica*

**DOI:** 10.3389/fmicb.2021.730355

**Published:** 2021-12-02

**Authors:** Kolathuru Puttamadaiah Ramesha, Nagabhushana Chandra Mohana, Siddaiah Chandra Nayaka, Sreedharamurthy Satish

**Affiliations:** ^1^Microbial Drug Technological Laboratory, Department of Studies in Microbiology, University of Mysore, Manasagangotri, Mysore, India; ^2^Vijnan Bhavan, University of Mysore, Manasagangotri, Mysore, India

**Keywords:** endophyte, epigenetic modification, *Nigrospora sphaerica*, HDAC, DNMT

## Abstract

During plant interaction, endophytes provide benefits to the host plant. Endophytes also contribute a variety of structural attributes with biological potential. *Nigrospora sphaerica*, which produces phomalactone from *Adiantum philippense* L., was subjected to epigenetic modification. High-performance liquid chromatography (HPLC) and Gas chromatography-mass spectrometry (GCMS) analysis were used to determine secondary metabolite profiling. Epigenetic modifiers like DNA Methyltransferase (DNMT) and Histone deacetylase (HDAC) inhibitors increased the expression of biosynthetic pathways. The activation of new metabolites was observed as a result of the activation of cryptic biosynthetic gene clusters, as well as the silencing of phomalactone in some treatments. When compared to DNMT treatments, HDAC treatments showed a significant increase in cryptic metabolite induction. The induction of cryptic metabolites with biological significance by HDAC treatment is supported by our findings.

## Introduction

Endophytes generate a variety of chemical skeletons with therapeutic use. Previous studies have found secondary metabolites as a result of evolutionary changes or mutation (Walton, 2000; De Felicio et al., 2012; Mohana et al., 2018; Ramesha et al., 2018). This aspect has been adapted by researchers to discover new molecular entities via artificial genetic variation induction strategies (Fischer et al., 2016). These variations, in turn, would activate new biosynthetic pathways, resulting in novel molecular entities. Several strategies have been proposed in this regard, including genome mining, epigenetic activation, combinatorial shuffling, and metabolomics (Mohana et al., 2018). The current study concentrated on epigenetic activation for the induction of cryptic biosynthetic gene clusters associated with the phomalactone-producing mycoendophyte *Nigrospora sphaerica* from *Adiantum philippense* L. Epigenetic induction methodologies are based on the aspects of chromatin condensation level variations exerted by epigenetic modifiers, which can either silence or induce changes in the metabolite profile (Gacek and Strauss, 2012). Previous reports suggest that putative biosynthetic gene clusters are located in the distal regions of the chromosome present in the heterochromatin state and are influenced by molecular mechanisms such as DNA methylation and histone deacetylation (Shwab et al., 2007; Gacek and Strauss, 2012). Secondary metabolite production is directly related to changes in methylation, histone, and acetylation. Biosynthetic genes in filamentous fungi are regularly organized in a cluster for secondary metabolite production pathways. In general, the secondary metabolic pathway, for which many gene clusters are responsible, remains silent and cryptic in laboratory conditions. These cryptic and silent genes can also be activated via epigenetic regulation (Rutledge and Challis, 2015). The previous survey revealed that endophytes can create significantly more analogs of prominence through epigenetic changes. These triggers have essentially been demonstrated to be an effective strategy for the expression and silencing of putative gene clusters for novel metabolites (Keller and Hohn, 1997). The possibility of inducing both components of epigenetic modulation, namely DNA methylation and histone modifications, was investigated. Histone deacetylase inhibitors like sodium butyrate, suberohydroxamic acid (SAHA), and valproic acid, as well as DNA methyltransferase inhibitors like 5-azacytidine and hydralazine hydrochloride, were used to study epigenetic modulation in the endophytic fungus *N. sphaerica*. The study discovered differences in secondary metabolite profiles when analyzed using thin layer chromatography (TLC), high-performance liquid chromatography (HPLC), and gas chromatography-mass spectrometry (GCMS) analyses, establishing the induction of cryptic pathways when fermented *in vitro*.

## Materials and Methods

### Isolation of Endophytic Fungi

*Adiantum philippense* L. asymptomatic leaf sample was taken from healthy plant. Within 24 h of collecting, the plant material wrapped in sterile polythene bags and brought to the lab, where it was processed. The plant material was first washed under tap water to remove undesirable debris and soil, then cleaned twice with sterile double distilled water (SDw). The plant leaf tissue was cut into 0.5 cm (De Felicio et al., 2012) segments. The plant segments were surface sterilized with 75 percent (v/v) ethanol for 1 min, followed by 4 percent (v/v) NaOCl for 4 min, and then 75 percent (v/v) ethanol for 1 min. Finally, leaf tissue fragments were blot dried under aseptic circumstances after being rinsed with sterile double distilled water to eliminate remaining surface sterilizing agents. To minimize bacterial growth chloramphenicol (100 mg/L) were supplemented into medium. In each Petri plate, ten segments were put in 20 mL PDA medium. The Petri plates were sealed with cling film and incubated at 27 ± 2°C for 2 weeks in a light chamber with 12-h light/dark cycles. Individual fungal colonies were picked from the edge with a sterile fine pointed needle and placed onto antibiotic-free potato dextrose agar (PDA) plates for 14 days of incubation. Endophytic fungal strains were identified using standard guidelines based on the appearance of the fungal culture colony or hyphae, the features of the spores, and reproductive structures and molecular affiliation. The isolates were preliminarily screened using agar plug assay for antimicrobial activity, followed by secondary screening (Mohana et al., 2020, 2021).

From the above bioprospection we identified *N. sphaerica* from *Adiantum philippense* L. in the Western Ghats region near Virajpete in prior antimicrobial profiling attempts for bioactive endophyte. *N. sphaerica* (GenBank Accession number: MF400860) was found to biosynthesize phomalactone (Ramesha et al., 2020). In this regard, we have investigated to understand the probable changes caused by epigenetic modulations.

### Epigenetic Modifiers Stock Solutions

Six epigenetic modifiers were opted based on literature review viz., two DNMT inhibitors 5-azacytidine (A2385, Sigma-Aldrich) and hydralazine hydrochloride (H1753, Sigma-Aldrich), a sirtuins activator [quercetin (Q4951, Sigma-Aldrich)] and three HDAC inhibitors Suberoylanilide Hydroxamic Acid (SAHA) (390585, Sigma-Aldrich), sodium butyrate (303410, Sigma-Aldrich) and valproic acid (P4543, Sigma-Aldrich)] (de la Cruz et al., 2012; González-Menéndez et al., 2016).

### Inoculum Preparation

The wild type fungus *N. sphaerica*, preincubated on PDB at 27 ± 2°C for 7 days. For each treatment, selected epigenetic modifier (5-azacytidine, hydralazine hydrochloride, quercetin, Suberoylanilide Hydroxamic Acid, sodium butyrate, and valproic acid) was dissolved in DMSO and aseptically added (100, 250, and 500 μM concentration) (De Felicio et al., 2012; de la Cruz et al., 2012).

### Epigenetic Modifiers Treatment and Fermentation

PDB (HiMedia) (10 mL) was used as the seed medium for inoculation. Inocula agar plugs containing mycelial of a fungal strain cultivated on PDA medium for 7 days at 27 ± 2°C were transferred onto sterilized 10 mL of PDB medium for the control inocula. To assure sheared hyphae and mycelial disc fragments for homogeneous hyphal suspensions, the flasks were incubated on an orbital shaker (200 rpm). PDB was prepared for each epigenetic modifier treatment, and each epigenetic modifier was dissolved in DMSO and aseptically poured to each flask to achieve a final concentration of 100, 250, and 500 μM. The flasks were then incubated at 27 ± 2°C for 14 days. For extracting mycelium, the broth culture was filtered through a double-layered sterile muslin cloth. The culture filtrate was then extracted with ethyl acetate and dried for TLC and HPLC analysis to detect changes in secondary metabolites (de la Cruz et al., 2012; González-Menéndez et al., 2016).

### Preliminary Thin Layer Chromatography Profiling

To study the effect of the epigenetic modifier, 100, 250, and 500 μM of 5-azacytidine, Suberoylanilide Hydroxamic Acid, Hydralazine Hydrochloride, Sodium butyrate, Valproic Acid, and Quercetin was supplemented in 10 mL PDB broth with control flask containing only PDB. Later inoculated with *N. sphaerica* and incubated at 27 ± 2°C under shaking conditions. Following fermentation and crude ethyl extracts were profiled using TLC in solvent system of ethyl acetate and hexane (1:1) (Ramesha et al., 2020).

### Spectral Measurements

#### High-Performance Liquid Chromatography Profiling of Crude Extract

HPLC analysis of crude extract was done with Shimadzu UFLC—LC—20AD (5 μm, C18, 250 × 4.6 mm LC) with injection volume and flow rate of 20 μL and 1.0 mL/min, respectively, using methanol and absorbance measured at 254 nm wavelength.

#### Gas Chromatography-Mass Spectrometry Analysis

GCMS analysis of ethyl acetate extracts were carried out by Shimadzu GC-MS (Model Number: QP2010S; Rxi-5Sil MS) and comparison of compounds using the NIST 11 library.

### Antibacterial Activity

#### Test Organisms for Antibacterial Assay

The test bacterial cultures were procured from Microbial Type Culture Collection (MTCC) viz., Gram-positive bacteria *Staphylococcus aureus* (MTCC 7443), and Gram-negative *Escherichia coli* (MTCC 7410) were grown on MHA and used for the antibacterial assay.

#### Disc Diffusion Method

The disc diffusion method was used to determine the antibacterial activity of epigenetic modifier treated endophytic fungal extract. Sterile media plates were seeded with predetermined test microbial inocula (McFarland standard) as described by Mohana et al. (2020) on MHA. Sterile discs (6 mm) impregnated with 8 μL (40 μg/disc) of crude extract, were placed on the surface of the respective agar media using sterile forceps on sterile media plates with gentamicin (10 μg/disc) as positive control, sterile discs with 8 μL pure ethyl acetate served as negative control. Inoculated plates were incubated at 37 ± 2°C for 24 h. After the incubation period, the diameter of the zone of inhibition was measured around discs in mm. The assay was performed in triplicates and mean diameters were recorded (Mohana et al., 2020, 2021).

### Statistical Analysis

Data from three replicates were analyzed for each experiment and analysis of variance (ANOVA) using SPSS Inc., 16.0 for bioactive compound phomalactone. Significant effects of treatments were determined by *F*-values (*p* ≤ 0.05). Tukey’s HSD test separated treatment means.

## Results

### Preliminary Thin Layer Chromatography Profiling of Epigenetic Modifier Treatment of *Nigrospora sphaerica*

**FIGURE 1:**
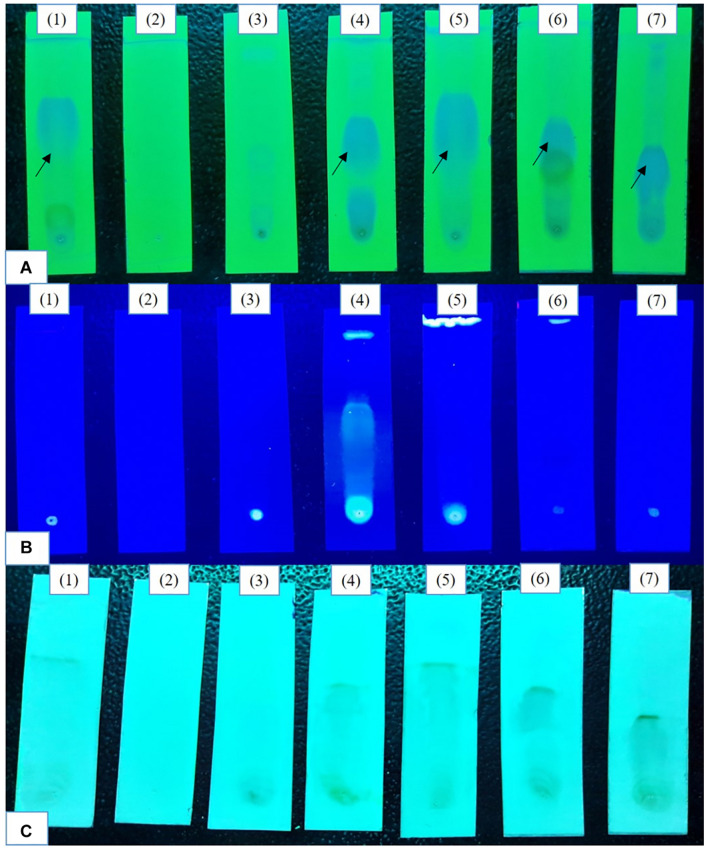
Comparative analysis of the TLC profiles produced by the strain with and without the epigenetic modifier treatment (500 μM) **(A)** 254 nm (short UV); **(B)** 365 nm (long UV); **(C)** TLC sprayed with chromophore agent vanillin. Control (1), 5-azacytidine (2), Valproic acid (3), Hydralazine Hydrochloride (4), Sodium butyrate (5), Quercetin (6), and SAHA (7).

Initial epigenetic modifier treatments (100, 250 μM) there was no visible induction of cryptic metabolites in TLC profiles (Supplementary Figure 1). At 500 μM a bright spot representing phomalactone (*R*_*f*_ = 4.5) (Supplementary Figure 2) was observed in all treatments except 5-azacytidine and valproic acid treatments. The intensity of the spot was brightest after 14 days of fermentation. In 365 nm Sodium butyrate treatment exhibited compounds. Vanillin chromophore spray agent revealed brownish black spots in control, sodium butyrate, quercetin and SAHA treatments confirm the presence of aldehydes and ketones which were absent in 5-azacytidine, valproic acid, hydralazine hydrochloride treatments (Figure 1).

### High-Performance Liquid Chromatography Analysis of Crude Epigenetic Modifiers Treated Extracts

**FIGURE 2:**
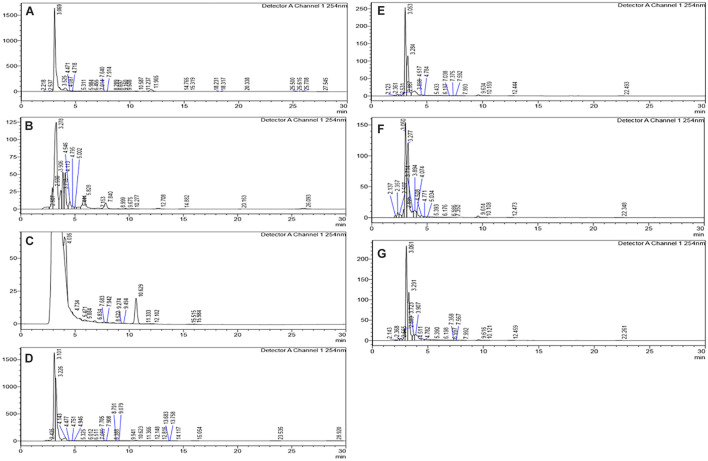
Comparative analysis of the differential HPLC secondary metabolite profiles produced by the strain with and without the epigenetic modifier treatment. Control **(A)**, 5-azacytidine **(B)**, Valproic acid **(C)**, Hydralazine Hydrochloride **(D)**, Sodium butyrate **(E)**, Quercetin **(F)**, and SAHA **(G)**.

The HPLC analyses of crude extracts derived from differently treated cultures revealed that the treatment of *N. sphaerica* by epigenetic modifiers had induced various cryptic compounds that were not observed in case of control. The analysis revealed 5-azacytidine treated culture produced 22 compounds, SAHA produced 24 compounds, Hydralazine Hydrochloride produced 27 compounds, Sodium butyrate produced 27 compounds, Valproic Acid produced 15 compounds and Quercetin produced 25 compounds and control had 29 compounds. The induction of cryptic metabolites was significant with 5-azacytidine (11), Suberoylanilide Hydroxamic Acid (19), Hydralazine Hydrochloride (19), Sodium butyrate (22), Valproic Acid (10) and Quercetin (18). The number of common compounds present in the treated, as well as control samples, were detected more in the culture treated with 5-azacytidine (11), Suberoylanilide Hydroxamic Acid (05), Hydralazine Hydrochloride (08), Sodium butyrate (05), Valproic Acid (05) and Quercetin (07) (Figure 2, Table 1, and Supplementary Table 1).

**TABLE 1:** Variation of secondary metabolites based on the HPLC profile from different epigenetic modifier treatments.

	**Control**	**5-Azacytidine**	**Valproic acid**	**Hydralazine Hydrochloride**	**Sodium butyrate**	**Quercetin**	**SAHA**
Total number of compounds detected	29	20	15	27	19	20	20
Number of common compounds detected in treated and control		02	04	05	05	05	05
Number of cryptic compounds detected in treated cultures		18	11	22	14	15	15
Number of compounds missing in treated cultures		27	25	24	24	24	24

### Gas Chromatography-Mass Spectrometry Analysis

The GCMS analysis revealed a total of 21 volatile organic compounds (VOCs) in control without epigenetic treatments. The highest number of VOCs were observed in case sodium butyrate treatments followed by hydralazine hydrochloride. The number of similar VOCs which were retained after treatment was highest in SAHA followed by sodium butyrate, hydralazine hydrochloride (Table 2, Supplementary Table 2, and Supplementary Figures 3–9).

**TABLE 2:** Volatile organic secondary metabolites detected by GCMS analysis from different epigenetic modifier treatments.

	**Control**	**5-Azacytidine**	**Valproic acid**	**Hydralazine Hydrochloride**	**Sodium butyrate**	**Quercetin**	**SAHA**
Total number of compounds detected	21	07	03	21	24	05	15
Number of common compounds detected in treated and control		01	00	06	08	00	09
Number of cryptic compounds detected in treated cultures		06	03	15	16	05	06
Number of compounds missing in treated cultures		20	21	15	13	21	12

### Antibacterial Activity by Disc Diffusion Method of Epigenetic Modifier Treated Extracts

**FIGURE 3:**
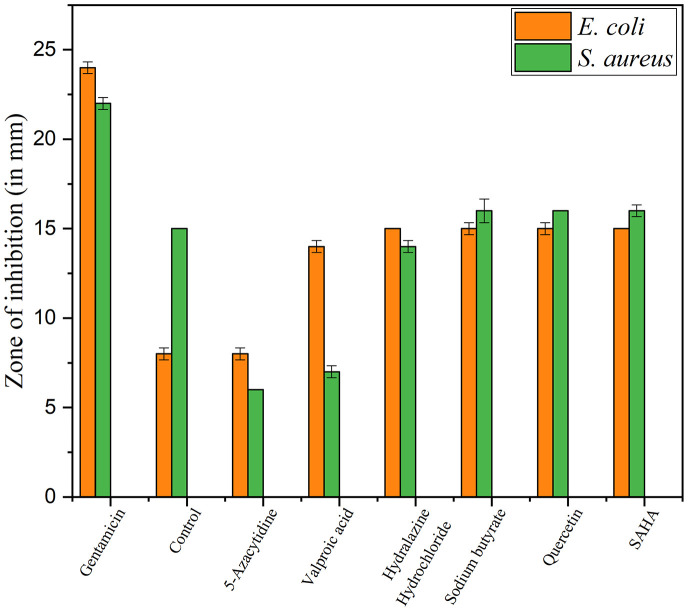
Antibacterial activity values of Disc diffusion assay for epigenetic treated crude extract.

**FIGURE 4:**
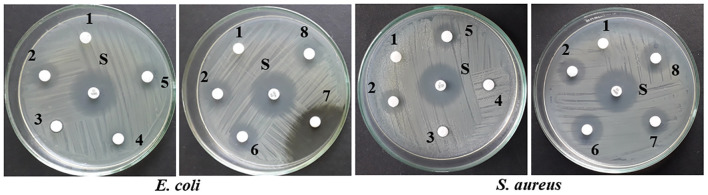
Antibacterial activity of crude EtOAc extract isolated from treated and untreated control cultures of *N. sphaerica*. Negative Control (1), Control without treatment (2), 5-azacytidine (3), Valproic acid (4), Hydralazine Hydrochloride (5), Sodium butyrate (6), Quercetin (7), and SAHA (8).

Upon epigenetic treatment the bioactive isolate had lost antimicrobial efficacy for 5 azacytidine and valproic acid whereas increased inhibition was observed for hydralazine hydrochloride and quercetin treatments, epigenetic modifiers sodium butyrate and SAHA had no visible changes for inhibition against Gram-negative *E. coli* for control. In the case of *S. aureus*, epigenetic treatment with hydralazine hydrochloride had retained its antibacterial, and the zone of inhibition was similar to control extract without any treatment (Figures 3, 4 and Supplementary Table 3). The enhanced zone of inhibition from the treatments could be attributed to cryptic compounds induced bearing antimicrobial activity along with phomalactone.

## Discussion

Previously, epigenetic research was limited to developmental and clinical biology, but in the last decade, it has drawn attention from other disciplines, opening the door to new possibilities. For novel structural entities with biological activity, an epigenetic strategy has been established in the search for natural products from microorganisms (Palmer and Keller, 2010; Kumar et al., 2016). In the current investigation, Phomalactone producing *N. sphaerica* and epigenetic induction was used to activate the bioactive cryptic metabolites. Both DNA methylation and histone deacetylase (HDAC) inhibitors were found to be successful in inducing the endophytic fungus *N. sphaerica* for cryptic metabolites, however, silencing of genes was detected in most treatments, as well as an increase in the yield of the bioactive metabolite phomalactone. In the case of filamentous fungus, the cryptic putative biosynthetic gene clusters are heterochromatin (De Felicio et al., 2012; Gacek and Strauss, 2012). The usage of HDACs causes histone hypoacetylation by removing acetyl groups from histone amino tails, resulting in a significant reduction in DNA methylation within the fungal genome, affecting global transcriptional regulatory mechanisms. Chemical profiling was used to assess the cryptic metabolites induced, along with HPLC. When compared to the control, the HDAC treatments demonstrated a high rate of cryptic metabolite induction as well as a high number of missing metabolites. SAHA treatment resulted in the production of 19 cryptic chemicals.

The sodium butyrate treatment resulted in the highest induction rate of 22 cryptic metabolites, as well as the retention of 5 comparable compounds produced in the control. Valproic acid exhibited the lowest induction of cryptic metabolites among the epigenetic treatments tested, with 10 cryptic metabolites induction followed by 5 comparable chemicals in control with suppression of 24 secondary metabolites. Similar findings were obtained when Valproic acid was applied to the *Phomopsis longicolla* strain for the synthesis of dicerandrol (De Felicio et al., 2012). The quercetin treatment caused a total of 18 compounds, with 7 of them being present in the control. In the case of HDAC inhibition, a “position effect” was seen, as well as the induction of new metabolites, resulting in total repression of dormant genes (Locke et al., 1988; Wilson et al., 1990). *Phoma* sp. demonstrated the existence of previously known (10’S)-verruculide B, vermistatin, and dihydrovermistatin (Gubiani et al., 2017) when treated with Suberoylanilide hydroxamic acid (SAHA). DNMT inhibitors reduce DNA-methylation by silencing genes, which affects developmental and other cellular processes, resulting in novel phenotypic traits (Deepika et al., 2016; Mohana et al., 2018). 5-Azacytidine treatment resulted in the induction of 11 cryptic chemicals. When compared to the control, 5-Azacytidine inhibited the expression of a total of 18 compounds. Hydralazine Hydrochloride, an epigenetic modifier, generated 19 cryptic metabolites with 8 comparable molecules when compared to the control, but reduced 24 metabolites after treatment. The GC-MS identification revealed presence of VOCs 3,5-Di-t-butylphenol; 2-Isopropyl-5-methyl-1-heptanol; 2-tert-Butyl-4,6-bis (3,5-di-tert-butyl-4 hydroxybenzyl) phenol; 2-Isopropyl-5-methyl-1-heptanol; Pentadecane; Dioctyl phthalate; 2-Isopropyl-5-methyl-1-heptanol previously reported with bioactivities such as antimicrobial, anti-biofilm and radical scavenging (Table 3).

**TABLE 3:** Volatile organic secondary metabolites reported for biological activity.

**Sl. No.**	**Cryptic compound**	**Bioactivity**	**References**
**5-Azacytidine**			
1	3,5-Di-t-butylphenol	Antibiofilm and antifungal activity	Rathna et al., 2016
2	2-Isopropyl-5-methyl-1-heptanol	Antimicrobial activity	Mannaa and Kim, 2018
3	2-tert-Butyl-4,6-bis (3,5-di-tert-butyl-4 hydroxybenzyl) phenol	Radical scavenging activity	Rao and Naika, 2018
**Valproic acid**			
1	2-tert-Butyl-4,6-bis (3,5-di-tert-butyl-4 hydroxybenzyl) phenol	Radical scavenging activity	Rao and Naika, 2018
**Hydralazine hydrochloride**			
1	2-Isopropyl-5-methyl-1-heptanol	Antimicrobial activity	Mannaa and Kim, 2018
2	Pentadecane	Antimicrobial activity	Bruno et al., 2015
3	Dioctyl phthalate	Antibacterial activity	Sastry and Rao, 1995
4	Squalene	Antimicrobial and anti-oxidative activity	Biswas and Chakraborty, 2013
**Sodium butyrate**			
1	Pentadecane	Antimicrobial activity	Bruno et al., 2015
2	Squalene	Antimicrobial and anti-oxidative activity	Biswas and Chakraborty, 2013
**Quercetin**			
1	Pentadecane	Antimicrobial activity	Bruno et al., 2015

In the present study to evaluate the epigenetic effect of chemical modifiers (HDACs and DNMTs) to induce genes that are latent under normal conditions was investigated. Among epigenetic modifiers employed, sodium butyrate and Hydralazine Hydrochloride, decreased PKS gene expression in the bioactive fungus *Nigrospora sphaerica*, as proven by spectroscopic analytical techniques. Furthermore, a molecular method based on PKS, NRPS, terpene synthase, or dimethylallyl tryptophan synthase would provide insights into BGCs and their prediction (Cacho et al., 2015).

## Conclusion

The present study successfully established the epigenetic induction with cryptic bioactive metabolite achieved. The elicitation and cryptic compound induction were prominent with sodium butyrate and hydralazine hydrochloride treatments along with enhancement of antimicrobial efficacy by phomalactone. However, the evaluation of the whole genome for all the treatments could envision epigenetic modulation causing the variations which need to be elucidated further. The present study had been focused only on antimicrobial efficacy by epigenetic treatments further studies on various bioactivities such as antioxidant, anticancer, etc. would be investigated in the future. The need for new analogs for various preclinical requisites of modern medicine would be envisioned with epigenetic strategies. The molecular-level changes exerted by epigenetic treatment would aid in mechanisms and pathways involved. The attempt of taking advantage of the biosynthetic potential of bioactive isolate understudy has been explored.

## Data Availability Statement

The original contributions presented in the study are included in the article/Supplementary Material, further inquiries can be directed to the corresponding author/s.

## Author Contributions

KPR and SS designed the concept and prepared the manuscript. KPR executed the experiments and data analysis. NCM assisted in the experiment, data analysis, preparation of manuscript along with proof correction. SCN helped in analysis, revision and proof correction. All authors have read and approved the manuscript.

## Conflict of Interest

The authors declare that the research was conducted in the absence of any commercial or financial relationships that could be construed as a potential conflict of interest.

## Publisher’s Note

All claims expressed in this article are solely those of the authors and do not necessarily represent those of their affiliated organizations, or those of the publisher, the editors and the reviewers. Any product that may be evaluated in this article, or claim that may be made by its manufacturer, is not guaranteed or endorsed by the publisher.
