# Supplementary material for: Epigenetic Modifiers Revamp Secondary Metabolite Production in Endophytic Nigrospora sphaerica
Source: Front Microbiol. 2021 Dec 2;12:730355. doi: 10.3389/fmicb.2021.730355 (PMC8678036; doi:10.3389/fmicb.2021.730355)
Supplement: Supplementary file 1 [file Data_Sheet_1.docx]

Supplementary Material

# Supplementary Data


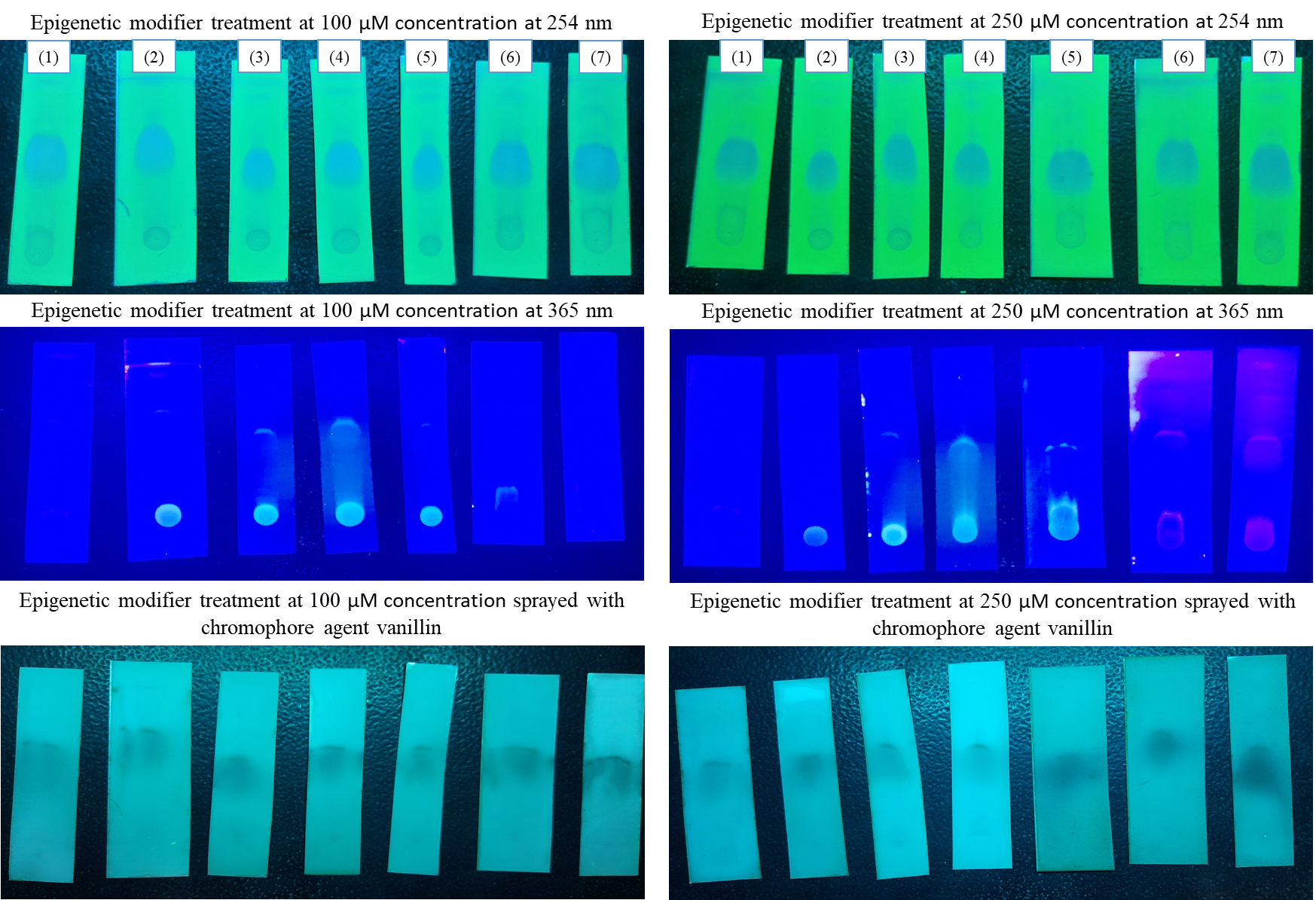


**Figure S1.** Comparative analysis of the TLC profiles produced by the strain with and without the epigenetic modifier treatment (1), 5-azacytidine (2), Valproic acid (3), Hydralazine Hydrochloride (4), Sodium butyrate (5), Quercetin (6) and SAHA (7).


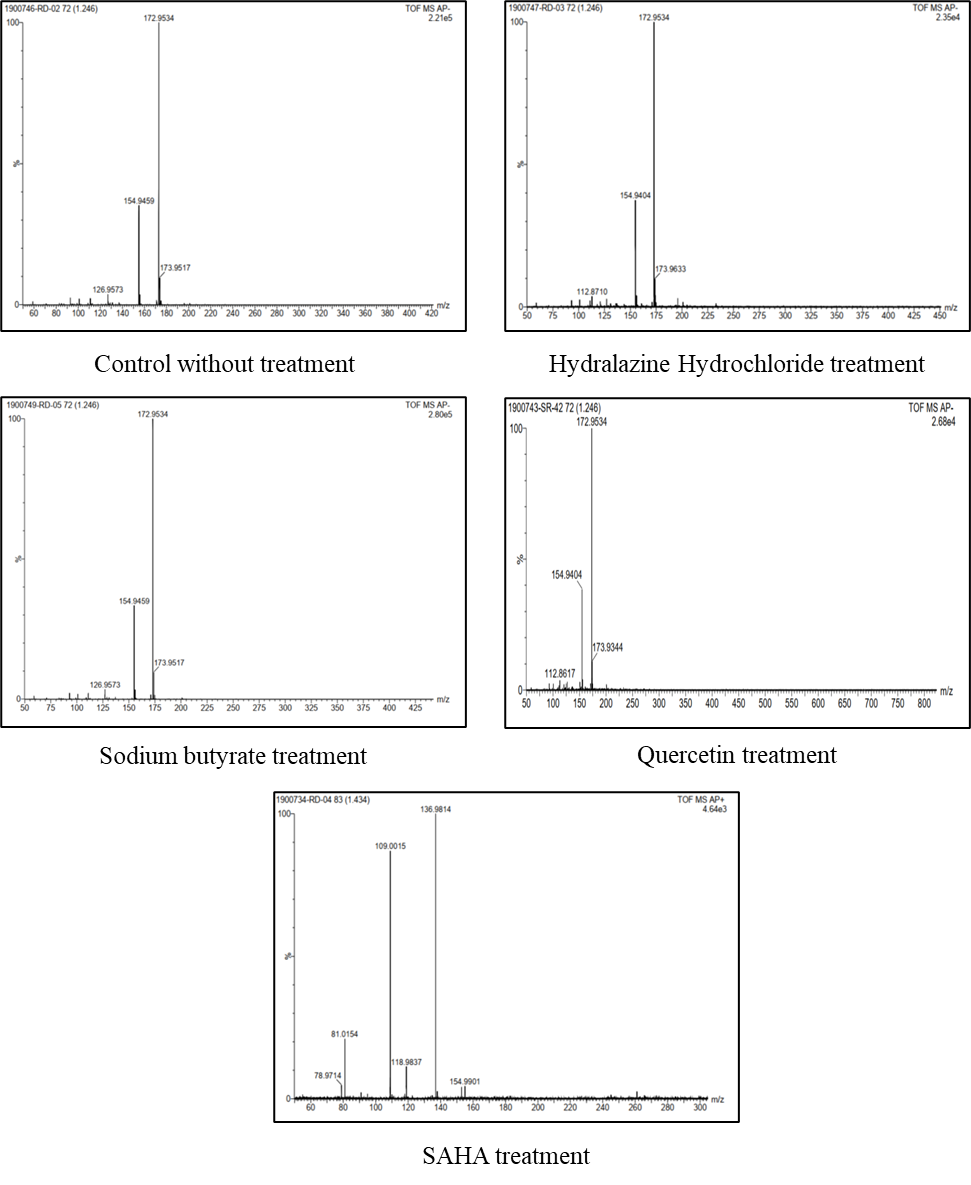


**Figure S2:** LCMS analysis of treatments showing phomalactone (*m/z*: 154).

**
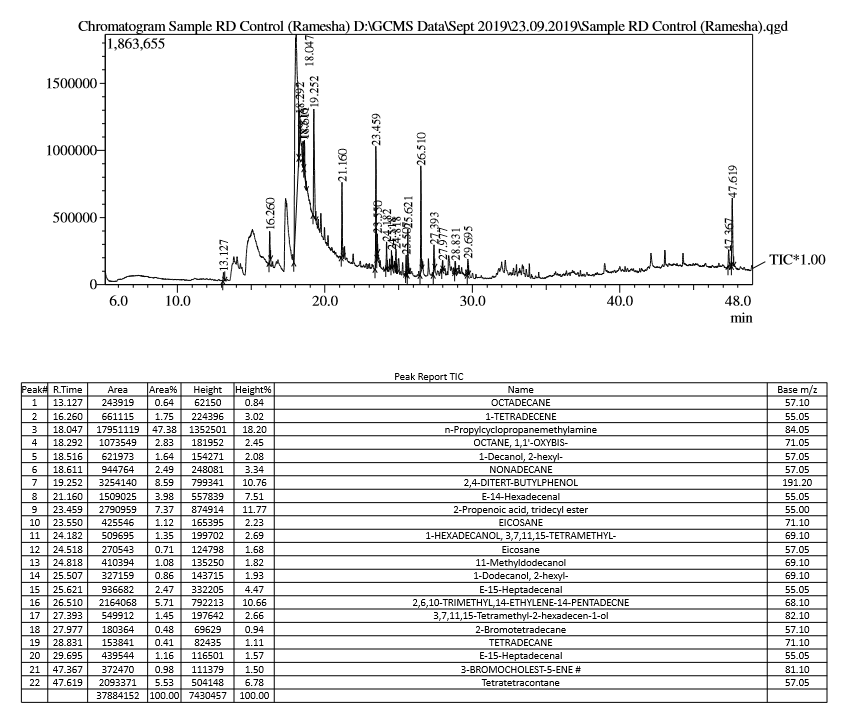
**

**Figure S3.** GCMS Data of Control crude extract without treatment

**
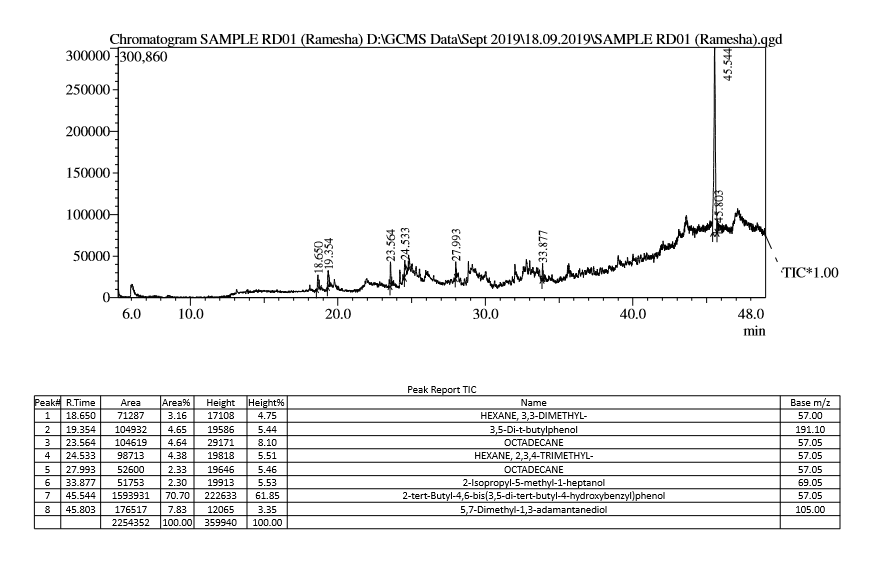
**

**Figure S4.** GCMS data of 5-Azacytidine treatment

**
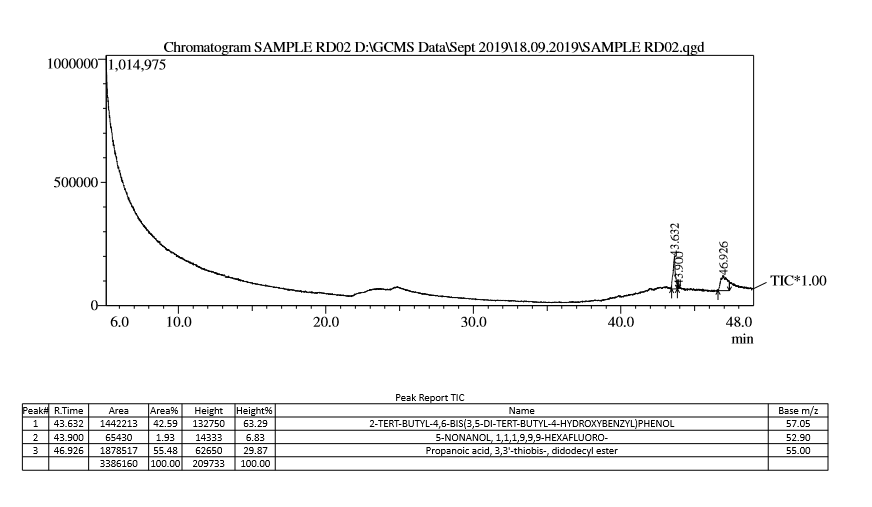
**

**Figure S5.** GCMS data of Valproic acid treatment

**
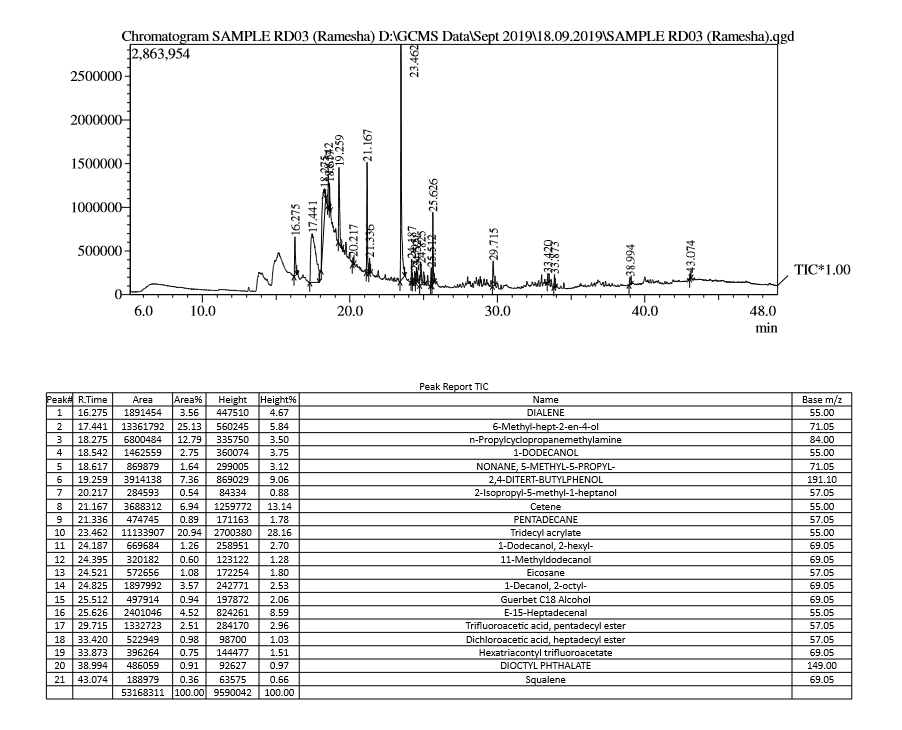
**

**Figure S6.** GCMS data of Hydralazine hydrochloride treatment

**
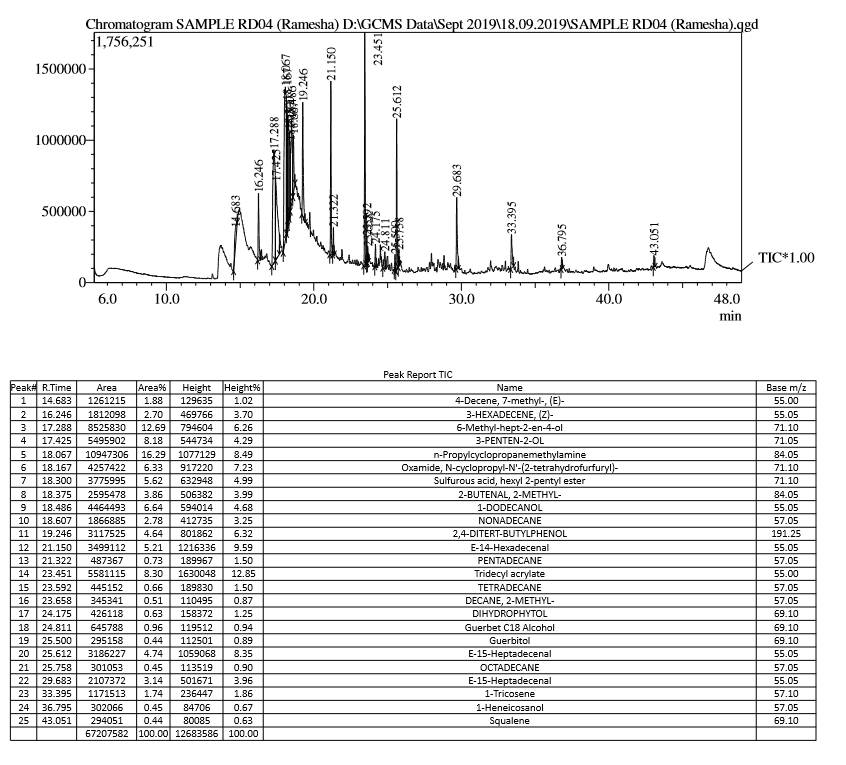
**

**Figure S7.** GCMS data of Sodium butyrate treatment

**
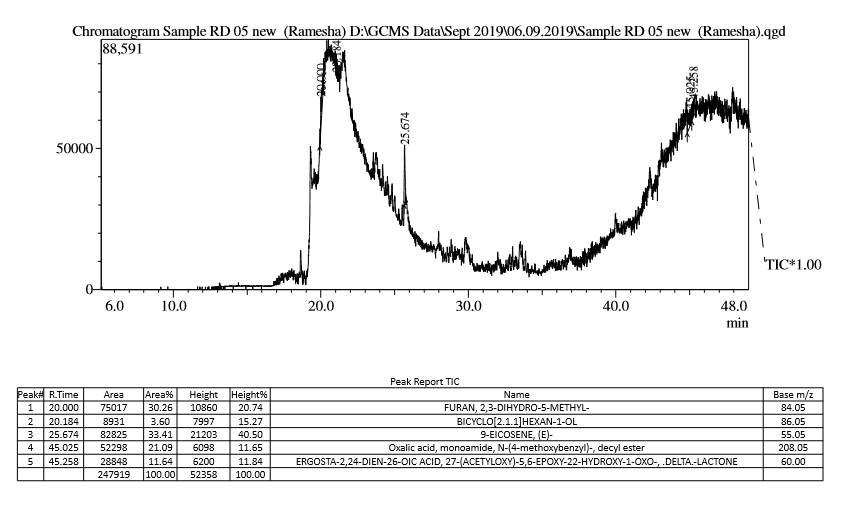
**

**Figure S8.** GCMS data of Quercetin treatment

**
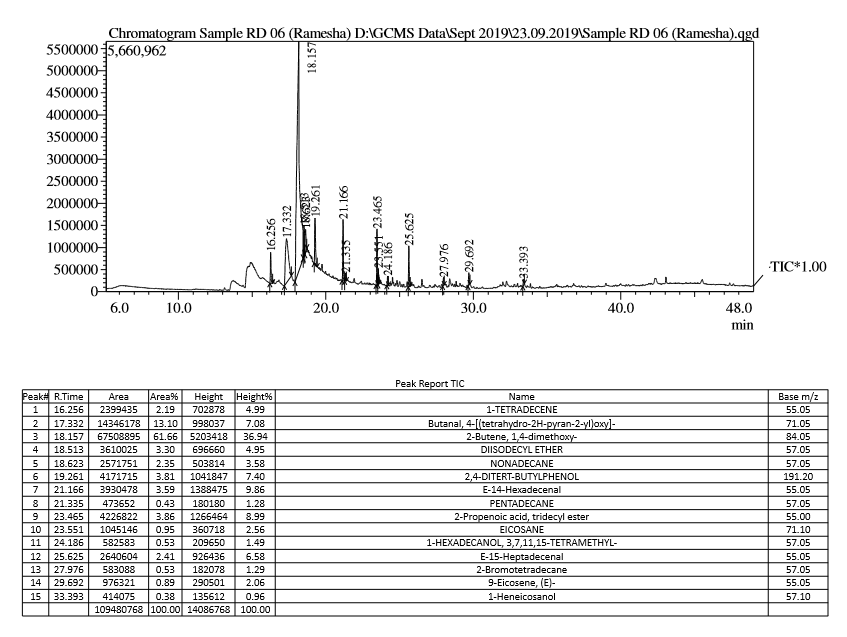
**

**Figure S9.** GCMS data of SAHA treatment

**Table S1.** Number of peaks detected in HPLC profiles of the total metabolites of *Nigrospora sphaerica* at wavelength of 254nm from different treatments

| Peak no. | Retention Time | Control | 5-azacytidine | Valproic acid | Hydralazine Hydrochloride | Sodium butyrate | Quercitin | SAHA |
| --- | --- | --- | --- | --- | --- | --- | --- | --- |
|  |  | **(Peak Area)** | **(Peak Area)** | **(Peak Area)** | **(Peak Area)** | **(Peak Area)** | **(Peak Area)** | **(Peak Area)** |
| 1 | 2.1 | 0 | **0** | **0** | **0** | 10278 | 30182 | 9850 |
| 2 | 2.2 | 51226 | 0 | 0 | 0 | 0 | 0 | 0 |
| 3 | 2.3 | 0 | 0 | 0 | 0 | 42678 | 80296 | 45871 |
| 4 | 2.4 | 0 | 0 | 0 | 167987 | 0 | 0 | 0 |
| 5 | 2.5 | 41091 | 93109 | 0 | 0 | 32448 | 65575 | 49559 |
| 6 | 2.8 | 0 | 0 | 0 | 0 | 57559 | 108057 | 167155 |
| 7 | 2.9 | 0 | 278406 | 0 | 0 | 0 | 0 | 0 |
| 8 | 3.0 | 20310377 | 0 | 0 | 0 | 2086152 | 1367324 | 1992990 |
| 9 | 3.1 | 0 | 0 | 0 | 15954884 | 0 | 0 | 0 |
| 10 | 3.2 | 0 | 2212928 | 0 | 11772237 | 1266280 | 1510219 | 1346813 |
| 11 | 3.5 | 78922 | 0 | 0 | 0 | 0 | 0 | 0 |
| 12 | 3.7 | 0 | 336629 | 0 | 0 | 0 | 7546 | 159678 |
| 13 | 3.8 | 0 | 0 | 0 | 0 | 404356 | 156345 | 0 |
| 14 | 3.9 | 0 | 478379 | 0 | 0 | 0 | 0 | 320309 |
| 15 | 4.0 | 806189 | 0 | 361746 | 0 | 0 | 103232 | 0 |
| 16 | 4.1 | 0 | 485952 | 0 | 1509254 | 0 | 0 | 0 |
| 17 | 4.4 | 6711 | 0 | 0 | 2544 | 0 | 0 | 0 |
| 18 | 4.5 | 0 | 143503 | 0 | 0 | 43713 | 45864 | 58565 |
| 19 | 4.7 | 6350 | 49004 | 4206 | 15560 | 40009 | 21755 | 69261 |
| 20 | 4.9 | 0 | 0 | 0 | 14289 | 0 | 0 | 0 |
| 21 | 5.0 | 0 | 30489 | 0 | 0 | 0 | 17085 | 0 |
| 22 | 5.3 | 27015 | 0 | 0 | 18683 | 0 | 33864 | 60526 |
| 23 | 5.4 | 0 | 60330 | 8681 | 0 | 38639 | 0 | 0 |
| 24 | 5.8 | 0 | 360436 | 13676 | 0 | 0 | 0 | 0 |
| 25 | 5.9 | 28064 | 0 | 0 | 0 | 0 | 0 | 0 |
| 26 | 6.0 | 0 | 0 | 0 | 41598 | 0 | 0 | 0 |
| 27 | 6.1 | 0 | 0 | 0 | 0 | 34596 | 22033 | 39806 |
| 28 | 6.4 | 26761 | 0 | 0 | 0 | 0 | 0 | 0 |
| 29 | 6.5 | 0 | 0 | 0 | 27615 | 0 | 0 | 0 |
| 30 | 6.8 | 0 | 0 | 13759 | 0 | 0 | 0 | 0 |
| 31 | 6.9 | 0 | 0 | 0 | 0 | 0 | 8079 | 16341 |
| 32 | 7.0 | 57927 | 0 | 0 | 94688 | 12600 | 0 | 0 |
| 33 | 7.1 | 0 | 43347 | 0 | 0 | 0 | 0 | 0 |
| 34 | 7.3 | 0 | 0 | 0 | 0 | 2962 | 1653 | 4575 |
| 35 | 7.5 | 0 | 0 | 0 | 0 | 3500 | 0 | 4573 |
| 36 | 7.6 | 20624 | 0 | 12428 | 0 | 0 | 0 | 0 |
| 37 | 7.7 | 0 | 0 | 0 | 31615 | 0 | 0 | 0 |
| 38 | 7.8 | 0 | 181192 | 0 | 0 | 0 | 0 | 0 |
| 39 | 7.9 | 15143 | 0 | 5521 | 23716 | 3918 | 0 | 3117 |
| 40 | 8.2 | 8366 | 0 | 0 | 0 | 0 | 0 | 0 |
| 41 | 8.3 | 0 | 0 | 0 | 19903 | 0 | 0 | 0 |
| 42 | 8.5 | 0 | 0 | 6924 | 0 | 0 | 0 | 0 |
| 43 | 8.6 | 5623 | 0 | 0 | 0 | 0 | 0 | 0 |
| 44 | 8.7 | 0 | 0 | 0 | 19099 | 0 | 0 | 0 |
| 45 | 8.9 | 0 | 2165 | 0 | 0 | 0 | 0 | 0 |
| 46 | 9.0 | 0 | 0 | 0 | 17929 | 0 | 0 | 0 |
| 47 | 9.1 | 12491 | 0 | 0 | 0 | 0 | 0 | 0 |
| 48 | 9.2 | 0 | 0 | 3129 | 0 | 0 | 0 | 0 |
| 49 | 9.4 | 0 | 0 | 5102 | 0 | 0 | 0 | 0 |
| 50 | 9.5 | 4016 | 0 | 0 | 0 | 0 | 0 | 0 |
| 51 | 9.6 | 0 | 5869 | 0 | 0 | 42303 | 35051 | 23522 |
| 52 | 9.9 | 0 | 0 | 0 | 1675 | 0 | 0 | 0 |
| 53 | 10.1 | 0 | 0 | 0 | 0 | 5335 | 3471 | 4560 |
| 54 | 10.2 | 0 | 7784 | 0 | 0 | 0 | 0 | 0 |
| 55 | 10.5 | 121062 | 0 | 0 | 0 | 0 | 0 | 0 |
| 56 | 10.6 | 0 | 0 | 289461 | 181031 | 0 | 0 | 0 |
| 57 | 11.2 | 9291 | 0 | 0 | 0 | 0 | 0 | 0 |
| 58 | 11.3 | 0 | 0 | 1397 | 34793 | 0 | 0 | 0 |
| 59 | 11.9 | 725846 | 0 | 0 | 0 | 0 | 0 | 0 |
| 60 | 12.1 | 0 | 0 | 7158 | 106064 | 0 | 0 | 0 |
| 61 | 12.4 | 0 | 0 | 0 | 0 | 2443 | 2476 | 2313 |
| 62 | 12.7 | 0 | 21860 | 0 | 0 | 0 | 0 | 0 |
| 63 | 12.8 | 0 | 0 | 0 | 13897 | 0 | 0 | 0 |
| 64 | 13.6 | 0 | 0 | 0 | 4574 | 0 | 0 | 0 |
| 65 | 13.7 | 0 | 0 | 0 | 2857 | 0 | 0 | 0 |
| 66 | 14.1 | 0 | 0 | 0 | 4671 | 0 | 0 | 0 |
| 67 | 14.7 | 1206 | 0 | 0 | 0 | 0 | 0 | 0 |
| 68 | 14.8 | 0 | 2324 | 0 | 0 | 0 | 0 | 0 |
| 69 | 15.3 | 4836 | 0 | 0 | 0 | 0 | 0 | 0 |
| 70 | 15.5 | 0 | 0 | 3073 | 0 | 0 | 0 | 0 |
| 71 | 15.9 | 0 | 0 | 7291 | 0 | 0 | 0 | 0 |
| 72 | 16.0 | 0 | 0 | 0 | 4042 | 0 | 0 | 0 |
| 73 | 18.2 | 3977 | 0 | 0 | 0 | 0 | 0 | 0 |
| 74 | 18.3 | 3802 | 0 | 0 | 0 | 0 | 0 | 0 |
| 75 | 20.1 | 0 | 1305 | 0 | 0 | 0 | 0 | 0 |
| 76 | 20.3 | 30942 | 0 | 0 | 0 | 0 | 0 | 0 |
| 77 | 22.2 | 0 | 0 | 0 | 0 | 0 | 0 | 4525 |
| 78 | 22.3 | 0 | 0 | 0 | 0 | 0 | 5864 | 0 |
| 79 | 22.4 | 0 | 0 | 0 | 0 | 3380 | 0 | 0 |
| 80 | 23.5 | 0 | 0 | 0 | 5255 | 0 | 0 | 0 |
| 81 | 25.5 | 6201 | 0 | 0 | 0 | 0 | 0 | 0 |
| 82 | 25.6 | 2185 | 0 | 0 | 0 | 0 | 0 | 0 |
| 83 | 25.7 | 7108 | 0 | 0 | 0 | 0 | 0 | 0 |
| 84 | 26.0 | 0 | 36156 | 0 | 0 | 0 | 0 | 0 |
| 85 | 27.5 | 4929 | 0 | 0 | 0 | 0 | 0 | 0 |
| 86 | 28.9 | 0 | 0 | 0 | 24561 | 0 | 0 | 0 |

**Table S2.** GCMS analysis for detection of VOCs

| **Sl No.** | **Control** | **5-Azacytidine** | **Valproic acid** | **Hydralazine Hydrochloride** | **Sodium butyrate** | **Quercetin** | **SAHA** |
| --- | --- | --- | --- | --- | --- | --- | --- |
| **1** | octadecane | hexane, 3,3-dimethyl- | 2-tert-butyl-4,6-bis(3,5-di-tert-butyl-4-hydroxybenzyl) phenol | dialene | 4-decene, 7-methyl-, (e)- | furan, 2,3-dihydro-5-methyl- | 1-tetradecene |
| **2** | 1-tetradecene | 3,5-di-t-butylphenol | 5-nonanol, 1,1,1,9,9,9-hexafluoro- | 6-methyl-hept-2-en-4-ol | 3-hexadecene, (z)- | bicyclo[2.1.1]hexan-1-ol | butanal, 4-[(tetrahydro-2h-pyran-2-yl) oxy]- |
| **3** | phomalactone | octadecane | propanoic acid, 3,3'-thiobis-, didodecyl ester | phomalactone | 6-methyl-hept-2-en-4-ol | 9-eicosene, (e)- | 2-butene, 1,4-dimethoxy- |
| **4** | octane, 1,1'-oxybis- | hexane, 2,3,4-trimethyl- |  | 1-dodecanol | 3-penten-2-ol | oxalic acid, monoamide, n-(4-methoxybenzyl)-, decyl ester | diisodecyl ether |
| **5** | 1-decanol, 2-hexyl- | 2-isopropyl-5-methyl-1-heptanol |  | nonane, 5-methyl-5-propyl- | phomalactone | ergosta-2,24-dien-26-oic acid, 27-(acetyloxy)-5,6-epoxy-22-hydroxy-1-oxo-, .delta.-lactone | nonadecane |
| **6** | nonadecane | 2-tert-butyl-4,6-bis(3,5-di-tert-butyl-4-hydroxybenzyl) phenol |  | 2,4-ditert-butylphenol | oxamide, n-cyclopropyl-n'-(2-tetrahydrofurfuryl)- |  | 2,4-ditert-butylphenol |
| **7** | 2,4-ditert-butylphenol | 5,7-dimethyl-1,3-adamantanediol |  | 2-isopropyl-5-methyl-1-heptanol | sulfurous acid, hexyl 2-pentyl ester |  | e-14-hexadecenal |
| **8** | e-14-hexadecenal |  |  | cetene | 2-butenal, 2-methyl- |  | pentadecane |
| **9** | 2-propenoic acid, tridecyl ester |  |  | pentadecane | 1-dodecanol |  | 2-propenoic acid, tridecyl ester |
| **10** | eicosane |  |  | tridecyl acrylate | nonadecane |  | eicosane |
| **11** | 1-hexadecanol, 3,7,11,15-tetramethyl- |  |  | 1-dodecanol, 2-hexyl- | 2,4-ditert-butylphenol |  | 1-hexadecanol, 3,7,11,15-tetramethyl- |
| **12** | 11-methyldodecanol |  |  | 11-methyldodecanol | e-14-hexadecenal |  | e-15-heptadecenal |
| **13** | 1-dodecanol, 2-hexyl- |  |  | eicosane | pentadecane |  | 2-bromotetradecane |
| **14** | e-15-heptadecenal |  |  | 1-decanol, 2-octyl- | tridecyl acrylate |  | 9-eicosene, (e)- |
| **15** | 2,6,10-trimethyl,14-ethylene-14-pentadecne |  |  | guerbet c18 alcohol | tetradecane |  | 1-heneicosanol |
| **16** | 3,7,11,15-tetramethyl-2-hexadecen-1-ol |  |  | e-15-heptadecenal | decane, 2-methyl- |  |  |
| **17** | 2-bromotetradecane |  |  | triﬂuoroace9c acid, pentadecyl ester | dihydrophytol |  |  |
| **18** | tetradecane |  |  | dichloroace9c acid, heptadecyl ester | guerbet c18 alcohol |  |  |
| **19** | e-15-heptadecenal |  |  | hexatriacontyl triﬂuoroacetate | guerbitol |  |  |
| **20** | 3-bromocholest-5-ene # |  |  | dioctyl phthalate | e-15-heptadecenal |  |  |
| **21** | tetratetracontane |  |  | squalene | octadecane |  |  |
| **22** |  |  |  |  | 1-tricosene |  |  |
| **23** |  |  |  |  | 1-heneicosanol |  |  |
| **24** |  |  |  |  | squalene |  |  |

**Table S3.** Antibacterial activity of Epigenetic Modifiers treated crude extracts by disc diffusion method

| **Sl. No.** | **Test Organism** | **Gentamicin** | **Epigenetic Modifiers treated crude extracts (Zone of inhibition in mm)** | | | | | | |
| --- | --- | --- | --- | --- | --- | --- | --- | --- | --- |
|  |  | In mm | Control | 5-Azacytidine | Valproic acid | Hydralazine Hydrochloride | Sodium butyrate | Quercetin | SAHA |
| 1. | *Escherichia coli* | 24 ± 0.33 | 8 ± 0.33 | 8 ± 0.33 | 14 ± 0.33 | 15 ± 0.00 | 15 ± 0.33 | 15 ± 0.33 | 15 ± 0.00 |
| 2. | *Staphylococcus aureus* | 22 ± 0.33 | 15 ± 0.00 | 6 ± 0.00 | 7 ± 0.33 | 14 ± 0.33 | 16 ± 0.66 | 16 ± 0.00 | 16 ± 0.33 |
